# Supplementary material for: Aerobic microbial life persists in oxic marine sediment as old as 101.5 million years
Source: Nat Commun. 2020 Jul 28;11:3626. doi: 10.1038/s41467-020-17330-1 (PMC7387439; doi:10.1038/s41467-020-17330-1)
Supplement: Supplementary file 1 — Supplementary Information [file 41467_2020_17330_MOESM1_ESM.pdf]

Supplementary Information

Aerobic microbial life that persists in oxic marine sediment for 101.5 million years

Yuki Morono[1,2], Motoo Ito[1,2], Tatsuhiko Hoshino[1,2], Takeshi Terada[3], Tomoyuki Hori[4], Minoru Ikehara[5], Steven D'Hondt[6], and Fumio Inagaki[1,2,7]*

[1]Kochi Institute for Core Sample Research, Japan Agency for Marine-Earth Science and Technology (JAMSTEC), 200 Otsu, Monobe, Nankoku, Kochi 783-8502, Japan; [2]Research and Development Center for Submarine Resources, JAMSTEC, 200 Otsu, Monobe, Nankoku, Kochi 783-8502, Japan; [3]Marine Works Japan Ltd, 3-54-1, Oppamahigashi, Yokosuka, Kanagawa 237-0063, Japan; [4]Environmental Management Research Institute, National Institute of Advanced Industrial Science and Technology (AIST), 16-1 Onogawa, Tsukuba, Ibaraki 305-8569, Japan; [5]Center for Advanced Marine Core research, Kochi University, Nankoku, Kochi, 783-8502, Japan; [6]Graduate School of Oceanography, University of Rhode Island, Narragansett Bay Campus, 215 South Ferry Road, Narragansett, RI 02882, USA; and [7]Research and Development Center for Ocean Drilling Science, 3173-25 Showa-machi, Kanazawa-ku, Yokohama, Kanagawa 236-0001, Japan
*present affiliation: Mantle Drilling Promotion Office (MDP), Institute for Marine-Earth Exploration and Engineering, JAMSTEC, Yokohama 236-0001, Japan

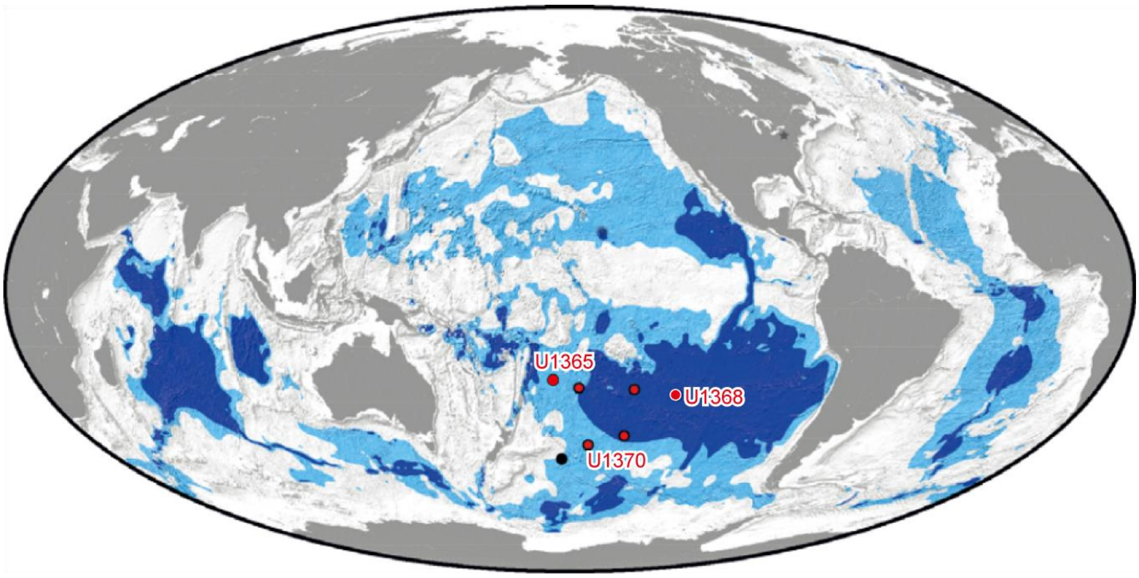

Supplementary Figure 1 | Map showing locations of sampling sites. Sites are plotted on a global map of regions with dissolved oxygen and aerobic activity throughout the entire sedimentary sequence[8]. Dark blue indicates minimum areal extent of these regions and light blue indicates maximum extent of these regions.

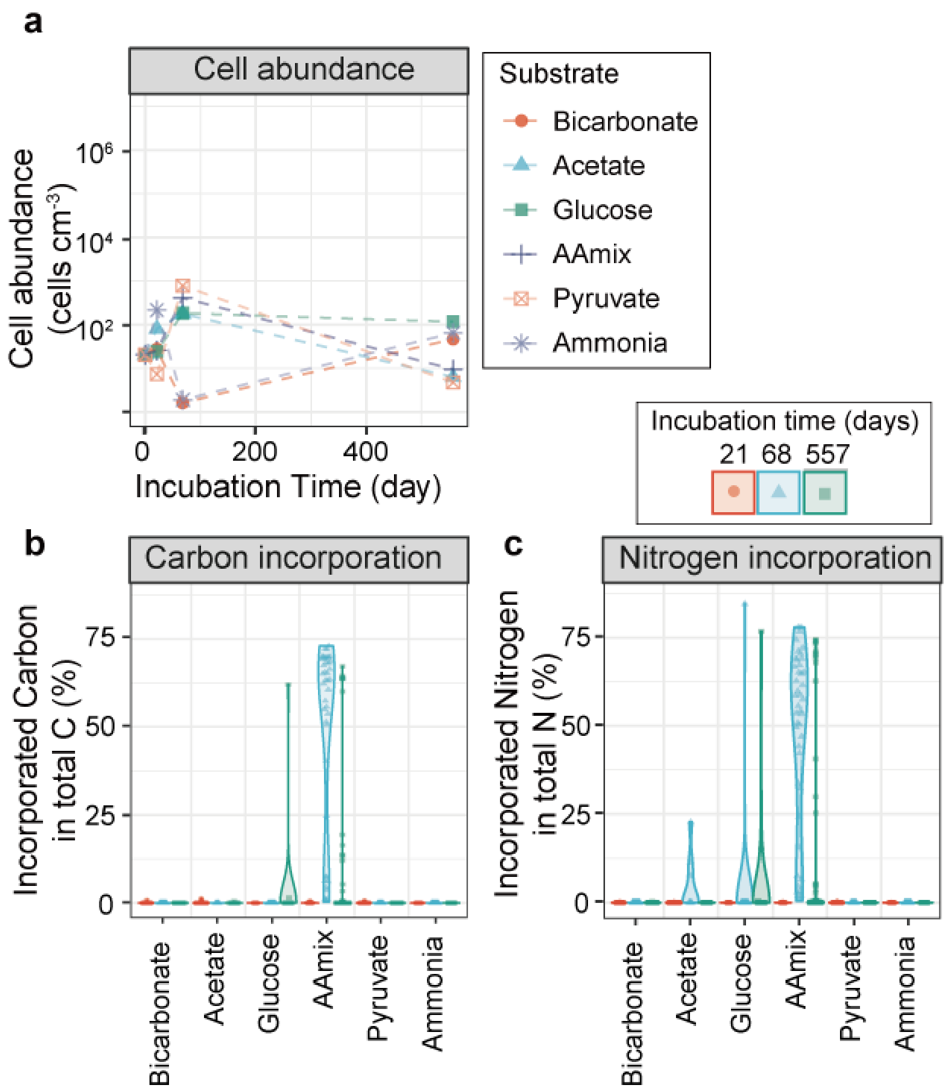

Supplementary Figure 2 | Microbial responses to the addition of carbon and nitrogen substrates for incubation set up for U1370 7H-2. a. Plot of cell abundance during incubation. Cell abundances for Incubation time 0 are abundances in the sediment samples before incubation was initiated. For the samples incubated with carbon substrates (bicarbonate, acetate, glucose, and pyruvate), ammonia was added as nitrogen source. The incubation labeled “Ammonia” received ammonia as the nitrogen source with no additional carbon. n = 19 samples (one for Incubation time 0 and three time points [days 21, 68 and 557] per substrate). **b, c.** Incorporation of carbon- (**b**) and nitrogen- (**c**) substrates by microbes identified by NanoSIMS cellular ROIs (Supplementary Data 1). Substrate incorporation for each ROI was plotted over kernel density violin plots.

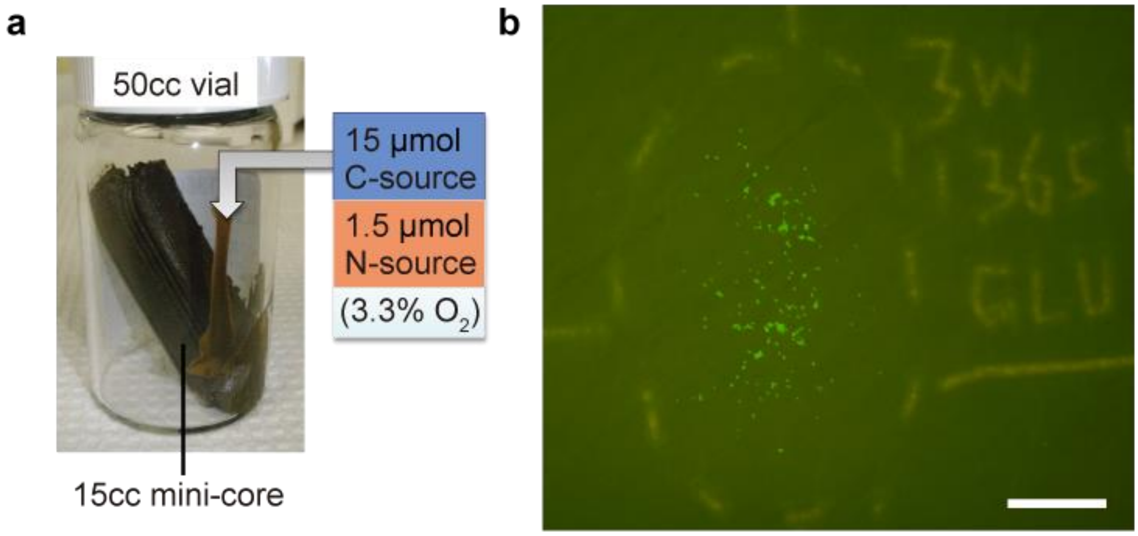

Supplementary Figure 3 | Incubation set up and NanoSIMS sample preparation. **a.** Sediment samples were incubated in vials with addition of carbon and nitrogen substrates under microaerobic condition (U1365 8H-2, 9H-3, U1368 1H-2, 2H-5) and anaerobic condition (U1370 7H-6). **b.** Example image of FAC-sorting of cells on ITO-coated polycarbonate membrane (U1365 9H-3 with ^{13}C-glucose and ^{15}N-ammonia as substrates). Bar represents 50 µm. Similar images were obtained for 95 samples processed in this study.
