# Supplementary material for: Aerobic microbial life persists in oxic marine sediment as old as 101.5 million years
Source: Nat Commun. 2020 Jul 28;11:3626. doi: 10.1038/s41467-020-17330-1 (PMC7387439; doi:10.1038/s41467-020-17330-1)
Supplement: Supplementary file 3 — Description of Additional Supplementary Information [file 41467_2020_17330_MOESM3_ESM.pdf]

Description of Additional Supplementary Files

File Name: Supplementary Data 1
Description: Substrate incorporation of each single cell. In total, 6986 cells were analyzed. Zero means no incorporation detected.

File Name: Supplementary Data 2
Description: OTUs and its phylogenetic classification table for the microbial cells separated and sorted from incubated sediments. The samples shown as incubation time zero are the sediment samples before incubation was initiated.
