# Supplementary material for: Aerobic microbial life persists in oxic marine sediment as old as 101.5 million years
Source: Nat Commun. 2020 Jul 28;11:3626. doi: 10.1038/s41467-020-17330-1 (PMC7387439; doi:10.1038/s41467-020-17330-1)
Supplement: Supplementary file 6 — Reporting Summary [file 41467_2020_17330_MOESM6_ESM.pdf]

nature research

Corresponding author(s): Yuki Morono
Steve D'Hondt
Fumio Inagaki

Last updated by author(s): Jun 8, 2020

Reporting Summary

Nature Research wishes to improve the reproducibility of the work that we publish. This form provides structure for consistency and transparency in reporting. For further information on Nature Research policies, see our Editorial Policies and the Editorial Policy Checklist.

Statistics

For all statistical analyses, confirm that the following items are present in the figure legend, table legend, main text, or Methods section.

n/a	Confirmed	
☐	☒	The exact sample size (*n*) for each experimental group/condition, given as a discrete number and unit of measurement
☐	☒	A statement on whether measurements were taken from distinct samples or whether the same sample was measured repeatedly
☐	☒	The statistical test(s) used AND whether they are one- or two-sided<br>*Only common tests should be described solely by name; describe more complex techniques in the Methods section.*
☐	☒	A description of all covariates tested
☐	☒	A description of any assumptions or corrections, such as tests of normality and adjustment for multiple comparisons
☐	☒	A full description of the statistical parameters including central tendency (e.g. means) or other basic estimates (e.g. regression coefficient) AND variation (e.g. standard deviation) or associated estimates of uncertainty (e.g. confidence intervals)
☐	☒	For null hypothesis testing, the test statistic (e.g. *F*, *t*, *r*) with confidence intervals, effect sizes, degrees of freedom and *P* value noted<br>*Give P values as exact values whenever suitable.*
☒	☐	For Bayesian analysis, information on the choice of priors and Markov chain Monte Carlo settings
☒	☐	For hierarchical and complex designs, identification of the appropriate level for tests and full reporting of outcomes
☒	☐	Estimates of effect sizes (e.g. Cohen's *d*, Pearson's *r*), indicating how they were calculated

Our web collection on statistics for biologists contains articles on many of the points above.

Software and code

Policy information about availability of computer code

Data collection	Microbial cells that incorporated stable isotope-labeled substrates were analyzed using NanoSIMS 50L with an analysis software of NanoSIMS operation software v4.3 (AMETEK Co. Ltd., CAMECA BU). Fluorescence images were acquired with either Olympus cellSens software v1.16 Standard or DP2-BSW v.2.1 (Olympus).
Data analysis	A description of the software and code has been included in the Methods. Isotope incorporation data analysis and display as violin plots of the kernel density function were done using CAMECA WinImage software (v.3.1.4), OpenMIMS plugin32 in ImageJ33 distribution of Fiji, and R (v.3.6.2) with the “ggplot2”, “cowplot”, “ggsci”, “scales” packages. The obtained sequence reads were quality trimmed and filtered by FASTQ<br>Toolkit in Basespase (v.2.2.5, Illumina). Resultant paired-end reads were processed and assigned taxonomy using mothur and the SSURef_NR99_123_SILVA database following the Mothur software package (v.1.35.0) of Illumina MiSeq Standard Operating Procedure. After that, the sequences were clustered into operational taxonomic units (OTUs) at 97% similarity using usearch 10.0 program. Potentially-contaminated OTUs that have significantly higher (p<0.05, ANOVA) abundance in the extraction negative controls than in the sediment samples were identified by using Qiime and removed from further analysis. Non-metric multidimensional scaling were performed with the metaMDS function of R software package vegan.

For manuscripts utilizing custom algorithms or software that are central to the research but not yet described in published literature, software must be made available to editors and reviewers. We strongly encourage code deposition in a community repository (e.g. GitHub). See the Nature Research guidelines for submitting code & software for further information.

Data

Policy information about availability of data

All manuscripts must include a data availability statement. This statement should provide the following information, where applicable:

- Accession codes, unique identifiers, or web links for publicly available datasets
- A list of figures that have associated raw data
- A description of any restrictions on data availability

DNA sequence data are deposited in the DNA Data Bank of Japan (DDBJ) under accession code of DRA007733. The authors declare that the data supporting the findings of this study are available within the paper and the Supplementary Information.

Field-specific reporting

Please select the one below that is the best fit for your research. If you are not sure, read the appropriate sections before making your selection.

- [x] Life sciences
- [ ] Behavioural & social sciences
- [ ] Ecological, evolutionary & environmental sciences

For a reference copy of the document with all sections, see nature.com/documents/nr-reporting-summary-flat.pdf

Life sciences study design

All studies must disclose on these points even when the disclosure is negative.

Sample size	To identify autotrophic and heterotrophic microbial populations, as well as their potential rates for growth and substrate uptake, sediment samples were incubated with stable isotope–labeled substrates (13C6-glucose, 13C2-acetate, 13C3-pyruvate, 13C-bicarbonate, 13C-15N-amino acids mix [mixture of 20 Amino Acids], and 15N-ammonium). The number of the incubations per sample vial was dependent on the estimated cell concentration, the number of substrates, time points, and control incubations. Given the estimated low concentration of microbial cells, the sample volume per incubation was maximized by sacrificing replicates. As a result, four replicates of incubation vials with 15 cm^3 mini-plugs (containing 1.5 × 10^3 to 4.4 × 10^4 cells per vial) were prepared for each substrate from 20 cm-long whole round core (corresponding to roughly 600 cm^3) sampled by avoiding outer edges of the sediment cores (which are likelier to be contaminated by drill fluid) and by taking an interior portion of a core with a sterile tip-cut 30 mL syringe. The number of cellular regions of interests (ROIs) analyzed per incubation sample ranged from 9 to 210, which was determined by the number of cells sorted for NanoSIMS analysis.
Data exclusions	No samples or analyses were excluded from the incubation experiments.
Replication	We prepared four replicates of incubation vials per each substrate, and one of each was sacrificed at three time points (21, 68, and 557 days of incubation). The low microbial abundance at the starting sediment (1.0 × 10^2 to 2.9 × 10^3 cells/cm^3-sediment) constrained the number of vials in the incubation. However, the consistent growth of microbes on each substrate confirmed that replicated experiment went successful.
Randomization	Prepared incubation vials were randomly assigned into experimental groups (incubation substrates, sampling time points).
Blinding	The incubation condition of the samples were coded into serial numbers, therefore, the investigators were blinded during data collection. The obtained data were aligned back to incubation conditions by the serial numbers after the analysis.

Reporting for specific materials, systems and methods

We require information from authors about some types of materials, experimental systems and methods used in many studies. Here, indicate whether each material, system or method listed is relevant to your study. If you are not sure if a list item applies to your research, read the appropriate section before selecting a response.

Materials & experimental systems

n/a	Involved in the study	
[x]	[ ]	Antibodies
[x]	[ ]	Eukaryotic cell lines
[x]	[ ]	Palaeontology and archaeology
[x]	[ ]	Animals and other organisms
[x]	[ ]	Human research participants
[x]	[ ]	Clinical data
[x]	[ ]	Dual use research of concern

Methods

n/a	Involved in the study	
[x]	[ ]	ChIP-seq
[x]	[ ]	Flow cytometry
[x]	[ ]	MRI-based neuroimaging
